# Roles of pRB in the Regulation of Nucleosome and Chromatin Structures

**DOI:** 10.1155/2016/5959721

**Published:** 2016-12-22

**Authors:** Chiharu Uchida

**Affiliations:** Advanced Research Facilities & Services, Preeminent Medical Photonics Education & Research Center, Hamamatsu University School of Medicine, 1-20-1 Handayama, Higashi-ku, Hamamatsu, Shizuoka 431-3192, Japan

## Abstract

Retinoblastoma protein (pRB) interacts with E2F and other protein factors to play a pivotal role in regulating the expression of target genes that induce cell cycle arrest, apoptosis, and differentiation. pRB controls the local promoter activity and has the ability to change the structure of nucleosomes and/or chromosomes via histone modification, epigenetic changes, chromatin remodeling, and chromosome organization. Functional inactivation of pRB perturbs these cellular events and causes dysregulated cell growth and chromosome instability, which are hallmarks of cancer cells. The role of pRB in regulation of nucleosome/chromatin structures has been shown to link to tumor suppression. This review focuses on the ability of pRB to control nucleosome/chromatin structures via physical interactions with histone modifiers and chromatin factors and describes cancer therapies based on targeting these protein factors.

## 1. Introduction

**Figure 1:**
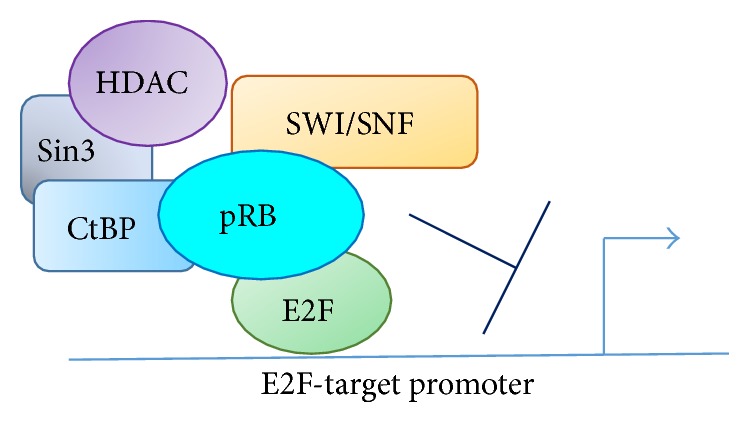
pRB blocks cell cycle progression by repressing the E2F-target gene transcription through the recruitment of transcriptional corepressors and/or chromatin remodeling protein factors, such as HDAC, Sin3, CtBP, and SWI/SNF, at promoter regions.

Retinoblastoma protein (pRB) was the first identified tumor suppressor that negatively regulates the G0/G1 to S phase transition of the cell cycle [1–4]. The most studied mechanism by which pRB negatively regulates the cell cycle progression involves the biding of pRB to E2F transcription factors (E2F1, E2F2, and E2F3a), which inhibits E2F-mediated expression of S phase-promoting genes, such as DNA polymerase, dihydrofolate reductase, and cdc2 [5–8]. pRB inhibits E2F transcriptional activity via a direct interaction with E2F; however, pRB also blocks cell cycle progression by repressing the target gene transcription through the recruitment of transcriptional corepressors and/or chromatin remodeling protein factors at promoter regions [9] (Figure 1). The repressors and protein factors that cooperatively participate in the pRB-mediated transient repression and silencing of the target genes include histone deacetylase (HDAC) [10, 11], replication factor C [12], ATPase subunit of the SWI/SNF complexes Brm and BRG1 (Brm-related gene 1) proteins [13, 14], DNA methyltransferase DNMT1 [11], and heterochromatin protein HP1 [15], which all belong to “LXCXE proteins” that possess the LXCXE-binding motif for pRB [16]. In addition to these LXCXE proteins, pRB interacts with many nuclear proteins independently of the LXCXE motif, such as histone methyl transferase Suv39h1 [15, 17], histone demethylase LSD1 [18], and histone demethylase RBP2 (KDM5A) [19, 20]. Through the physical interaction with these protein factors, pRB is involved in not only local gene promoter inactivation but also global epigenetic control of cellular senescence [21] and differentiation [22]. Furthermore, pRB was recently shown to play a role in DNA replication during the S phase and G2/M phases via interactions with regulator proteins for DNA replication [12, 23], chromatin condensation [24–27], and mitotic spindle formation [28]. Understandably, cellular events, such as G0/G1 maintenance, DNA replication, and mitosis progression, require drastic nuclear structural changes and chromosomal rearrangement. In fact, pRB plays an important role in chromosome dynamics and modulation of chromatin structure. For example, pRB depletion alters chromatin structure due to changes in epigenetic histone modifications, such as methylation and acetylation, which controls the status in G0/G1 cells [9] or heterochromatin region in the interphase cells [29, 30]. pRB depletion can also cause incomplete chromosomal condensation and segregation in mitosis [24–27]. Importantly, it has been demonstrated that the aberrant chromatin structure and chromosome arrangement caused by pRB inactivation are associated with chromosomal instability [25, 27, 31], which is a hallmark of human cancer cells. The focus of this review is to highlight the active role of pRB in chromatin/chromosome structure and stability. Indeed, this appears to be the most crucial aspect in the tumor suppressor ability of pRB.

## 2. pRB-Mediated Repression of Gene Transcription via Chromatin Structure Modification

### 2.1. Cooperative Function of Chromatin Remodeling Complex SWI/SNF with pRB

The SWI/SNF is a chromatin remodeling protein complex that participates in ATP-dependent histone exchange or removal of histones from DNA, thereby altering nucleosome structure and mobilizing higher-order formation of chromatin [32]. SWI/SNF-mediated structural changes of nucleosomes are involved in both activation and repression of gene transcription depending on components of the SWI/SNF complex. As an example of transactivation ability, a SWI/SNF subunit, BRG1, is necessary for* MAX* gene transcription, MAX-dependent prodifferentiation gene expression, and the subsequent suppression of lung cancer development [33]. In this case, the BRG1-containing SWI/SNF complex may facilitate gene transcription by enhancing the accessibility of transcriptional activator proteins to the* MAX *enhancer/promoter regions. It is also known that some SW1/SNF complexes containing Brm and/or BRG1 bind to pRB and repress transcription. The ATPases of SWI/SNF/Brm/BRG1 are involved in chromatin remodeling and the pRB-mediated inhibition of cell proliferation. pRB was reported to recruit Brm or BRG1 through their LXCXE domains, thereby repressing gene expression and effectively inducing cell cycle arrest [13, 14]. Although the LXCXE-dependent interaction between endogenous pRB and Brm/BRG1 is not fully confirmed, their cooperative function was identified in transcriptional inactivation mechanisms [34]. The cell lines C33A and A437 are deficient in both Brm and BRG1 and are resistant to active pRB-mediated cell cycle arrest; however, ectopic expression of either Brm or BRG1 restored cell cycle arrest [35, 36]. Brm is required for nuclease resistance at cyclin A promoter region [36]. Although it is not clear whether Brm and BRG1 can be included in the same SWI/SNF/pRB complex, pRB uses the ATPase activity of Brm or BRG1 to change nucleosome structures. This occurs in cooperation with histone deacetylases and/or histone demethylases (as described below) to produce compact and tight nucleosome structures and thus repression of target gene expression. Because Brm and BRG1 can interact with both pRB and E2F [37], these ATPase chromatin remodelers efficiently facilitate the formation of closed chromatin structures and the pRB-mediated repression of the E2F-target genes.

### 2.2. Cooperative Function of Histone and DNA Modifiers with pRB

Histone deacetylase 1 (HDAC1) is also an important pRB binding protein for the inhibition of gene expression. In addition to the direct inhibition of E2F-mediated transactivation, pRB also recruits HDAC1 to the DNA strands near the promoter region of the E2F-target gene [10]. A canonical LXCXE motif derived from a viral oncogene competed with the pRB-HDAC1 binding, which suggests that the interaction between pRB and HDAC1 is LXCXE motif dependent [16]. However, recent studies have indicated that pRB-HDAC1 interactions can be indirect because HDAC1 is found in Sin3 and CtBP/CtIP complexes, which are also pRB-interacting proteins [38, 39].

Histone acetylation opens the chromatin structure so that transcriptional activators can access the target promoter region and stimulate transcription. On the other hand, HDACs catalyze the removal of an acetyl group from lysine residues in histones and nonhistone target proteins. By reducing acetylation, HDACs facilitate the inactivation of gene expression, including pRB-mediated repression of E2F-target gene expression (Figure 1). A previous study showed that the levels of histone acetylation at the E2F-target, that is, the* cyclin E promoter*, are reduced when* cyclin E* is silenced; furthermore, the HDAC inhibitor trichostatin A inhibited the pRB-mediated inactivation of cyclin E expression [40].

These studies suggest that pRB regulates the local chromatin structure by recruiting HDAC1 to modulate the balance of histone acetylation levels, and HDAC inhibitors may compromise the tumor suppressive pRB-E2F axis. However, a number of studies showed that HDACs are overexpressed in many human cancer cells [41, 42]. Indeed, many HDAC inhibitors have been characterized as anticancer drugs that show great efficacy for cancer cell death [43, 44]. This may reflect the pRB-E2F-independent effect of HDACs on cell viability, or the inhibitors may exert a stronger effect on HDAC-suppressed E2F-dependent apoptotic signaling compared to E2F-dependent cell proliferation.

Histone methylation and demethylation are important modifications of nucleosome/chromatin modifications induced by pRB. pRB interacts with the histone methyltransferase, Suv39h1, which is mainly responsible for trimethylation of H3K9 (H3K9me3), although it can also catalyze dimethylation of H3K9 (H3K9me2) [45, 46]. H3K9me2/3 is recognized by heterochromatin protein HP1 through its N-terminal chromodomain. This interaction changes the neighboring nucleosome structure into a packed form that is transcriptionally inactive. Accordingly, H3K9me3 is known as a “repressive histone mark” [47–50]. Notably, both of H3K9 methylation and HDAC-mediated deacetylation are induced on nucleosome histones near cyclin E promoter region after pRB-mediated E2F inactivation [15], suggesting that pRB has the ability to alter local chromatin structure via Suv39h1, HP1, and HDAC. HP1 is a family of three subtypes (HP1*α*, HP1*β*, and HP1*γ*) and each HP1 subtype plays common and also distinct roles in human cells. HP1*α* is mainly located in heterochromatin, while HP1*β* and HP1*γ* are associated with both heterochromatin and euchromatin [51, 52]. HP1 binds to the N-terminus of Suv39h1 through its chromoshadow domain [53, 54]. In this context, HP1*β*- or HP1*γ*-bound pRB may repress the euchromatic local promoter region of* cyclin E* by recruiting Suv39h1. This induces heterochromatin formation by recruiting additional Suv39h1 molecules to methylate the neighboring nucleosomes and produce a tightly packed and inactivated promoter region. Consistently, HP1*β* was found at E2F-responsive promoter regions when pRB was activated to repress these promoters [55]. Furthermore, pRB-HP1*γ* interaction mediates silencing of E2F-target gene expression and heterochromatin formation during senescence [56]. pRB-HP1*γ*-H3K9me3 is also involved in gene silencing in adult cardiac myocytes, which permanently exit the cell cycle [57]. However, it is not clear whether pRB directly interacts with HP1*α* to repress the expression of E2F-target genes because HP1*α* was found to be preferentially phosphorylated in the G2/M phase and to bind to histone H3 modified with both K9me3 and phosphorylated serine 10 in mitotic chromosomes [58]. HDAC-mediated deacetylation could effectively induce methylation in target regions, because HDAC interacts with Suv39h1 [59] and Suv39h1 binds to HP1 [47–50, 53, 54]. Although Suv39h1 does not have the LXCXE motif, in contrast to HP1, excess LXCXE peptides compete with pRB to bind to these proteins [15, 60]. Thus, many LXCXE-dependent interactions between pRB and pRB binding proteins are important for the regulation of chromatin structure dynamics.

LSD1 [18, 61] and RBP2 [20, 62] are pRB-interacting histone demethylases that catalyze the removal of methyl groups from H3K4me1/2 and H3K4me3, respectively. Methylated H3K4 is an “active histone mark” because it is enriched at the actively transcribing promoter region. pRB binds to these demethylases in a LXCXE-independent manner and represses transcription by recruiting them to demethylate H3K4me1/2 and H3K4me3 on the pRB-target promoter region. pRB recruits LSD1 on the same promoter for E2F binding; however, pRB-E2F immunoprecipitates did not contain LSD1 even though E2F was precipitated with LSD1 [18]. Although the functional significance of LSD1 on pRB-dependent E2F inhibition of cell cycle progression is not clear, recent studies showed that LSD1 is a member of different subsets of repressor complexes, such as CoREST families [63]. Importantly, these repressor complexes include several chromatin remodeling proteins and positively contribute to cell differentiation and somatic cell reprogramming. It is likely that the pRB-LSD1-E2F interaction functions in these cellular events.

At the onset of cell differentiation, cell cycle-driving gene expression is silenced for the exit from the cell cycle. The active histone marks are removed, while the repressive marks are introduced in target nucleosomes near the cell cycle-driving genes. The H3K4me3 demethylase activity of RBP2 has also been demonstrated to contribute to regulating cell differentiation [20]. Studies using RBP2 RNAi in pRB-null cells showed that RBP2 inhibits pRB-mediated differentiation under certain conditions; however, RBP2 also shares common roles with pRB at the initial step of differentiation by repressing transcription of cell cycle-driving genes [64]. These observations suggest that the pRB-mediated H3K4me3 demethylases modulate the histone modification with repressive marks on the pRB-target gene promoters and alter the chromatin structure to induce differentiation.

In addition to histone methyltransferase and demethylases, pRB binds to DNA methyl transferase 1 (DNMT1), which associates with HDAC* in vivo* [11]. pRB forms a complex with E2F, DNMT1, and HDAC through the LXCXE motif to repress E2F-mediated transactivation [11, 65]. Based on a previous report, the methylation of pRB-E2F's target promoter DNA may enhance and spread the histone modulation near the promoter. Many studies have demonstrated that methylated DNA recruits HDAC to deacetylate histones, thus resulting in an efficient repression of transcription [11, 66–68]. Although E2F-bound reporter DNA was not methylated under experimental conditions, the E2F-binding domain within a CpG-rich region of the endogenous* RB* promoter is highly methylated in many types of human cancer cells [17, 69, 70]. Taken together, these pRB binding histone modifiers, DNA methyltransferases, and chromatin modifiers can promote the pRB-dependent regulation of gene expression by changing the chromatin structure to a repressive form near the pRB-E2F-target promoter.

## 3. pRB-Mediated Regulation of Higher-Order Chromatin Structures and Chromosomes

In addition to the regulation of local nucleosome structures at the pRB-E2F-target promoter region, pRB plays a pivotal role in maintaining whole chromosome dynamics, such as heterochromatin formation and mitotic chromosome segregation. Cells expressing mutant pRB that lacks the LXCXE-interacting domain show abnormal chromatin structures, including decondensed chromatin and display butterfly chromosomes [71]. These aberrant chromosomes fail to properly separate during anaphase. This role of pRB is closely linked to terminal differentiation, senescence, and chromosome stability. In this section, the protein factors that directly/indirectly bind to pRB are discussed with a focus on regulation of higher-order of chromatin/chromosome structures.

### 3.1. The Role of pRB in Heterochromatin Formation

pRB participates in the formation and maintenance of heterochromatin structure [9, 72]. As described above, pRB binds to Suv39h and members of HP1 family, and the Suv39h-H3K9me3-HP1 axis is a key axis of regulator of heterochromatin formation [15, 45–60, 71]. In addition to H3K9m3, pRB binds to Suv4-20h1 and h2, which are methyltransferases that trimethylate histone H4K20 [71].

The H4K20me3 is enriched at pericentromeric heterochromatin, whereas pRB-deficient mouse fibroblasts show reduced levels of H4K20me3 at pericentromeric heterochromatin [73]. Similarly, cells that expressed a mutant pRB lacking the LXCXE-interacting domain (RB1^Δ*L*/Δ*L*^) showed diminished methylation of H4K20 at pericentromeric DNA [74]. Furthermore, loss of all of RB families caused a reduction in H4K20m3 levels at telomere DNA [75]. Interestingly, HP1 recruitment by the Suv39h-H3K9me3 axis is essential for Suv4-20h1/h2-mediated H4K20 trimethylation [76]. Notably, BRG1 depletion resulted in an aberrant chromatin organization caused by a dispersion of H3K9me3 and H4K20me3 and an increased mitotic failure caused by lagging anaphase chromosomes [77]. These effects are similar to the results found after pRB depletion in fibroblast cells. Taken together, these data strongly suggest that the regulation of type-specific histone methylation/demethylation by pRB leads to proper chromatin organization via several chromatin modulators, including HP1 and BRG1.

Polycomb group (PcG) proteins were originally identified as repressor complexes for* Hox *genes. PcG proteins regulate the Hox expression pattern required for development [78, 79]. Recent studies showed that PcG proteins are essential for the regulation of normal gene expression during cell differentiation and embryonic development [80, 81]. Two major PcG protein complexes, PRC1 and PRC2, are recruited to target sites in the genome [82] to modulate the chromatin structure and repress gene expression. Early studies revealed that HPC2, a PcG protein, coimmunoprecipitated with pRB, E2F, and CtBP and colocalized with pRB in a nuclear PcG complex in cultured cells [83]. In addition, pRB showed HPC2-dependent and HDAC-independent repressor activity for E2F-taget cyclin A gene expression [83]. pRB is required for the binding of PRC2 and its target gene to establish H3K27me3 at the gene site [84]. A recent study showed that RBR, a pRB ortholog in plants, directly interacts with PRC2 and inactivates the late embryonic genes through facilitating PRC2-mediated H3K27 trimethylation [85]. Thus, pRB promotes global gene silencing via interactions with PRC1 and PRC2, which contribute to cell differentiation and embryonic development.

### 3.2. The Role of pRB in Chromatin Structure and Dynamics for Differentiation and Senescence

**Figure 2:**
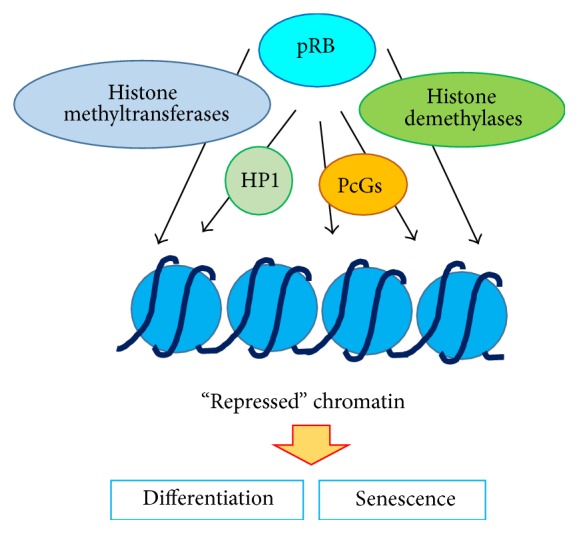
pRB facilitates cell cycle arrest and thus influences differentiation and senescence via interactions with histone modifiers and chromatin associating factors including histone methyltransferases, histone demethylases, HP1, and PcGs.

pRB facilitates cell cycle arrest and thus influences differentiation and senescence [21, 86] (Figure 2). Since differentiation requires multiple steps, including exit from the cell cycle and drastic changes in gene expression/silencing via both local and global nucleosome remodeling, notably, pRB binding epigenetic/chromatin modifiers are actively involved in differentiation. As described above, epigenetic or chromatin modifiers, such as histone demethylase RBP2 and the PcG protein complexes PRCs, are closely associated with pRB-mediated cell differentiation. One example is the role of these proteins in the pRB-meditated onset of myogenic differentiation [87, 88]. RBP2 appears to possess two opposing activities in pRB-mediated myogenic differentiation: inhibiting E2F-targeted cell cycle genes and the other is antagonizing differentiation by repressing the mitochondrial genes necessary for myogenic differentiation [89]. Apart from RBP2, Suv39h depletion in myoblasts leads to a reduction in H3K9 methylation, repression of S phase genes, and expression of myogenic marker genes under differentiating conditions [90].

Cellular senescence can be triggered by repetitive replication (replicative senescence), activation of oncogenic genes (oncogenic senescence), telomere shortening, and genotoxic stresses [91]. Senescence requires permanent cell cycle arrest and maintenance of a “repressed” nucleosome/chromatin structure. Here, the pRB-dependent packed nucleosome/chromatin structure appears to be a key event for the initiation and maintenance of senescence (Figure 2). Acute loss of pRB in senescent fibroblasts shows cell cycle reentry and recovery of cell proliferation [92], while reintroduction and overexpression of pRB in cancer cells induce senescence [93]. Furthermore, pRB is enriched on the E2F-target promoter region when cells are senescent [21, 56]. These observations prompt us to predict an active role of pRB in establishing senescence by forming a “repressed” chromatin structure. However, a previous study suggested that pRB plays a crucial role in the later stages of establishing or maintaining senescence, since cells lacking pRB or expressing a mutant pRB retain abilities to exhibit cell cycle arrest but definitively reenter to cell cycle and restart proliferation [94]. Thus, pRB-dependent epigenetic modification, that is, a repressive histone methylation mark, appears to be important for the establishment and maintenance of senescence. Indeed, pRB is necessary for the enrichment of H3K9me3 and demethylation of H3K4me3 on E2F-target promoters in senescent cells [56, 94, 95]. In addition, H3K9me3 levels are reduced in mutant pRB-expressing* RB1* ^Δ*L*/Δ*L*^ MEFs, which are unable to maintain senescence [94]. The study on* RB1* ^Δ*L*/Δ*L*^ MEFs also showed that the pRB binds to promyelocytic leukemia (PML) protein, and the LXCXE-interacting domain in pRB was important for PML-pRB binding to establish constitutive heterochromatin H3K9me3 at E2F-target genes [96–98]. Importantly, recent findings revealed that pRB was involved in the formation of senescent-associated heterochromatin foci (SAHF) [56]. This result furthers our understanding of the role of pRB in the establishment of senescence. SAHF is involved in the compaction of entire individual chromosomes and contain enriched H3K9me3, H3K27me3, and high mobility group A (HMGA) proteins that are known chromatin architectural factors. Active hypophosphorylated pRB is required for SAHF formation, and the knockdown of pRB inhibited SAHF formation [99–101]. Similarly, an experiment using E7-drived inactivation of pRB showed that pRB is crucial for HMGA2-induced SAHF formation [102]. pRB associates with PML to enrich H3K9me3 at the target genes, and PML can be a component of SAHF [96]. Taken together, pRB can control the structural changes in heterochromatin that are dependent on senescence induction, including SAHF formation; however, the precise mechanism by which pRB contributes to SAHF assembly remains unclear.

### 3.3. The Role of pRB in Chromatin Condensation and Chromosome Segregation

Early studies demonstrated that pRB is a component of nuclear matrix, which consists of highly compartmentalized and insoluble nonchromatin structures [103]. The nuclear matrix is composed of fibrogranular-like networks that associate with particular DNA regions and corresponding proteins. Thus, the matrix is considered as a platform where “DNA events” occur efficiently, such as transcription, replication, or heterochromatin formation, chromatin condensation, and chromatin remodeling. This indicates a crucial and primordial role for pRB as a nuclear matrix protein that actively participates in the repression of transcription and chromatin organization. A number of nuclear matrix proteins have been identified, including nuclear restricted protein/brain NRP/B, which binds to pRB and regulates neuronal differentiation [104]. This study suggests that an adequate composition of nuclear matrix proteins is important for cell function and pRB-dependent.

**Figure 3:**
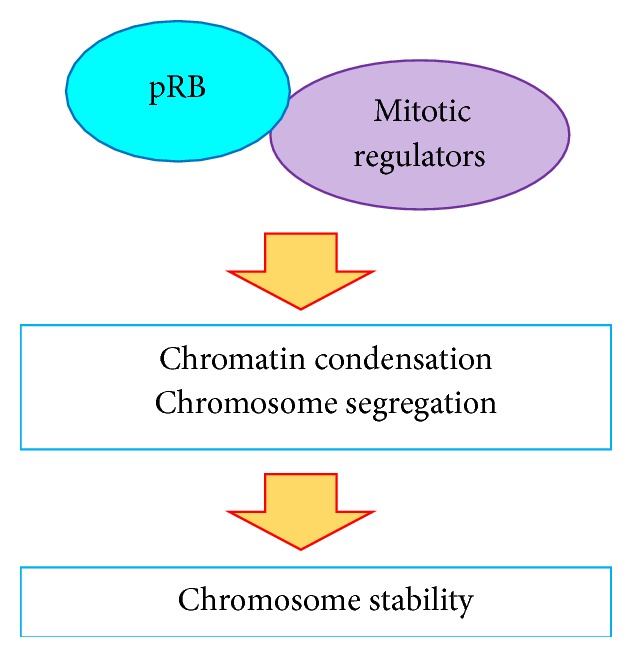
pRB is involved in proper chromatin condensation and chromosome segregation via interactions with mitotic regulators, such as condensin II and NuMA, which is important for chromosome stability.

Recently, pRB was discovered to bind to nuclear matrix apparatus protein NuMA [28], a mitotic spindle organizer and essential protein for mitotic progression [105]. Mitotic progression requires highly dynamic chromosome changes. Knockdown of pRB results in the aberrant distribution of NuMA in M phase cells and misalignment of spindle poles and spindle microtubules. Cells overexpressing mutant NuMA, which are deficient in pRB binding, showed similar defects. Notably, these M phase defects were associated with an uncondensed and dispersed chromosome structure, which can trigger chromosomal/genomic instability. Chromosomal instability is a hallmark of cancer cells accompanied with aneuploidy and an abnormal number of chromosomes, mainly caused by chromosome missegregation [106]. Importantly, a number of studies showed that pRB inactivation increased chromosomal instability [25–27, 107]. Consistently, the mutant NuMA-expressing cells showed low survival rates, and the surviving mutant cells showed multiple micronuclei after a long culture period [28]. These data indicate that the pRB-NuMA interaction is required for proper mitotic progression and chromosome organization (Figure 3).

Condensin II complex is another important factor that highlights the role of pRB in mitotic chromosome dynamics and stability (Figure 3). An initial study reported that Rbf, the fruit fly ortholog of pRB, interacted with drosophila condensin II subunit Cap-D3, which requires Rbf for the correct localization on chromosomes; furthermore,* Rbf* mutant showed abnormal and dispersed chromatin during prophase and prometaphase [24]. Additionally, human CAP-D3 (hCAP-D3) binds to pRb in an LXCXE-dependent manner, and* RB1* ^Δ*L*/Δ*L*^ cells displayed an inefficient localization of condensin II on chromosomes, delayed progression to metaphase, and lagging chromosomes in anaphase [26]. Moreover, a recent study showed that pRB, E2F, and hCAP-D3 form a complex at pericentromeric heterochromatin, and disruption of the complex in* RB1* ^−/−^ cells and* RB1* ^Δ*L*/Δ*L*^ cells correlated with an increase in aberrant replication, mitotic errors, and aneuploidy [27]. Surprisingly, the loss of even one copy of* RB1* can produce the same phenotype, suggesting that pRB plays a pivotal role in the maintenance of the chromosome structure and stability via physical interactions with chromatin-related proteins.

## 4. Maintenance of Nucleosome/Chromosome Structures by pRB and Cancer

pRB acts as a central tumor suppressor mainly by inhibiting the cell cycle progression driven by E2F-target genes. In this context, the involvement of pRB directs antitumorigenesis via a conformational change in the local promoter region with or without epigenetic marks. In many types of human cancer cells, the levels of pRB and pRB binding nucleosome/chromatin-related proteins that act cooperatively with pRB, such as HDACs [42], PML [98], and BRG1 [108], are decreased. Furthermore, loss of Suv4-20h1 in breast cancer cells was reported [109]. On the other hand, the binding proteins that are largely inactivated by pRB appear to be overexpressed in cancer cells. One example is that the expression of the H3K4me3 demethylase RBP2 was increased in lung cancer [110]. Interestingly, the H3K4me3 demethylase LSD1 is also overexpressed in many human cancers, including lung, breast, prostate, and blood cancers [63], which seems incomprehensible since LSD1 is a member of the pRB repressor complex. Some reports have proposed the tumor suppressor role of LSD1; however, the majority of studies demonstrated the tumor-promoting activity of LSD1 [111]. Although this controversial function needs to be fully investigated, it is possible that LSD1 plays two opposing roles that are dependent on the formation of distinct complexes. In support of this notion, LSD1 is able to act as a transcriptional activator and a repressor [112, 113]. A possible explanation is that LSD1 binds to the tumor suppressor, p53, to repress p53-mediated transcriptional activation and inhibit p53-induced apoptosis by removing monomethylation (K370me1) at K370 [114]. This indicates a tumor-promoting function of LSD1.

The increase in chromosomal instability due to dysfunctional pRB binding may be related to cancer development in the light of the normal pRB role in maintaining the global nucleosome structure and chromosome organization. Indeed, NuMA is overexpressed in colorectal and breast cancer [105, 115–118], suggesting that overexpressed NuMA, which can overcome sequestering by pRB, induces mitotic defects leading to chromosomal instability, which is similar to the results of pRB depletion. In addition, RB1^Δ*L*/Δ*L*^, a mutant pRB lacking the LXCXE-binding cleft, enhances tumorigenesis and genomic instability in mouse tumor models [26]. All of these findings support a central role for pRB and its nuclear binding proteins in the regulation and maintenance of the global nucleosome/chromosome structure, which is crucial for tumor suppression.

## 5. Cancer Treatments and Perspectives

Overall, this review focuses on the physical contribution of pRB, which controls local nucleosome structure and whole chromosome organization. pRB inactivation results in dysregulated cell proliferation, apoptosis, differentiation, and senescence, and all those defects can lead to tumorigenesis and cancer progression [31]. Phosphorylation is a well-known mechanism to inactivate pRB; in addition, pRB inactivation by oncogenic proteins is induced with viral infection, and* RB1* gene expression is repressed via promoter DNA methylation. The proteasome-dependent degradation of pRB promoted by ubiquitin ligase Mdm2, which was the first identified ubiquitin ligase for p53 [119, 120], is another pathway for pRB inactivation [121–126]. Therefore, inhibiting pRB inactivation is a relevant strategy to suppress cancer progression. Some efficacious compounds and small molecules have been investigated, such as CDK4/6 inhibitors to suppress pRB phosphorylation [127] and Nutlin-3, a small molecule inhibitor of Mdm2, to regulate Mdm2-mediated regulation of pRB expression [128–130]. The CDK4/6 inhibitor palbociclib is currently in phase II development, and ribociclib and abemaciclib are in phase I development. These inhibitors are being tested in breast cancer, lung cancer, liposarcoma, and neuroblastoma [131]. A recent study showed that Nutlin-3 caused p53 and p21 accumulation and hypophosphorylation of pRB, which lead to cell cycle arrest in some cell lines; however, in other cell lines, Nutlin-3 downregulated pRB and resulted in E2F-independent apoptosis [129]. These results are Mdm2-dependent, as evidenced by Mdm2 knockdown experiments that abolished the effects. Thus, Nutlin-3 is a potential therapeutic agent that can suppress and/or kill cancer cells. However, the mechanism by which Nutlin-3 induces degradation of hypophosphorylated pRB in some cells is not clear.

Targeting enzyme activities related to nucleosome histone modification may be a potent strategy for cancer therapy. Despite its repression of E2F-target gene expression, HDAC is overexpressed in many human cancers, and a number of HDAC inhibitors, including trichostatin A and vorinostat (also known as SAHA (suberoylanilide hydroxamic acid)), are antitumor agents [43]. SAHA was the first clinically approved HDAC inhibitor for the treatment of cutaneous T-cell lymphoma (CTCL). Belinostat (PXD101, Beleodaq) is used for the treatment of refractory peripheral T-cell lymphoma (PTCL), and panobinostat (LBH589) is used for the treatment of multiple myeloma. These drugs were approved by the FDA in 2014 and in 2015, respectively. In addition to these compounds, other HDAC inhibitors, including givinostat (ITF2357), abexinostat (PCI-24781), quisinostat (JNJ-26481585), resminostat (4SC-201), pracinostat (SB939), CUDC-101, CHR-2845, and CHR-2847, are currently in various clinical phases [43].

Chaetocin was the first discovered inhibitor for drosophila histone methyltransferase Su(var)3-9, and it selectively inhibits human Suv39h1 [111]. BIX01294 shows good* in vitro* inhibitory potency against Suv39h. LSD1 inhibitors, including the small molecules GSK2879552 and ORY1001, have been developed [63]. A screening of a panel of 165 cancer cell lines revealed that the SCLC and AML cell lines were sensitive to GSK2879552 [63]. Studies on the molecular mechanism of action suggested that GSK2879552 inhibits the demethylation of H3K4me1/2 by LSD1, leading to alterations in neuroendocrine gene expression and the suppression of SCLC cell growth. GSK2879552 is currently in a phase I clinical trial for AML and SCLC [132]. Compound 4SC-202 inhibits both HDAC1/2/3 and LSD1 and its phase I trial for the treatment of hematological tumors was recently completed [133].

Thus, the continued development of inhibitors of CDK4/6 and histone modifiers aims to eradicate cancer cells. Several agents showed sufficient potency in clinical trials. However, selective inhibitors or activators that target the interaction between pRB and its binding proteins during nucleosome/chromatin organization have not been identified. It is understandable that development of such agents, such as LXCXE-binding inhibitors, is difficult because pRB and its LXCXE-dependent interactions have central and diverse functions in living cells. To increase antitumor effectiveness, treatment with a combination of CDK4/6 inhibitors and inhibitors of histone modifiers could inhibit cell cycle progression and induce apoptosis via structural changes in the nucleosome/chromosome. A greater understanding of the direct role of pRB role in chromatin remodeling or chromosome organization will facilitate the development of antitumor agents and therapeutics for pRB-inactivated human cancers.
